# Teaching medical students a clinical approach to altered mental status: simulation enhances traditional curriculum

**DOI:** 10.3402/meo.v18i0.19775

**Published:** 2013-04-03

**Authors:** Jeremy D. Sperling, Sunday Clark, Yoon Kang

**Affiliations:** 1 Division of Emergency Medicine, Weill Cornell Medical College, New York, NY, USA; 2 Office of Academic Affairs and Department of Internal Medicine, Weill Cornell Medical College, New York, NY, USA

**Keywords:** mental status change, simulation, pre-clinical medical students

## Abstract

**Introduction:**

Simulation-based medical education (SBME) is increasingly being utilized for teaching clinical skills in undergraduate medical education. Studies have evaluated the impact of adding SBME to third- and fourth-year curriculum; however, very little research has assessed its efficacy for teaching clinical skills in pre-clerkship coursework. To measure the impact of a simulation exercise during a pre-clinical curriculum, a simulation session was added to a pre-clerkship course at our medical school where the clinical approach to altered mental status (AMS) is traditionally taught using a lecture and an interactive case-based session in a small group format. The objective was to measure simulation's impact on students’ knowledge acquisition, comfort, and perceived competence with regards to the AMS patient.

**Methods:**

AMS simulation exercises were added to the lecture and small group case sessions in June 2010 and 2011. Simulation sessions consisted of two clinical cases using a high-fidelity full-body simulator followed by a faculty debriefing after each case. Student participation in a simulation session was voluntary. Students who did and did not participate in a simulation session completed a post-test to assess knowledge and a survey to understand comfort and perceived competence in their approach to AMS.

**Results:**

A total of 154 students completed the post-test and survey and 65 (42%) attended a simulation session. Post-test scores were higher in students who attended a simulation session compared to those who did not (*p*<0.001). Students who participated in a simulation session were more comfortable in their overall approach to treating AMS patients (*p*=0.05). They were also more likely to state that they could articulate a differential diagnosis (*p*=0.03), know what initial diagnostic tests are needed (*p*=0.01), and understand what interventions are useful in the first few minutes (*p*=0.003). Students who participated in a simulation session were more likely to find the overall AMS curriculum useful (*p*<0.001).

**Conclusion:**

Students who participated in a simulation exercise performed better on a knowledge-based test and reported increased comfort and perceived competence in their clinical approach to AMS. SBME shows significant promise for teaching clinical skills to medical students during pre-clinical curriculum.

## Introduction

Simulation-based medical education (SBME) has become an increasingly common and potentially effective method for teaching clinical skills in both undergraduate (1, 2) and graduate medical education (1–4). Simulation as a teaching format provides a safe, supportive learning environment (5), can encourage the development of skills through experiential learning (6), and allows students to learn clinical skills through deliberate practice (7, 8). A large array of clinical situations can be realistically simulated using various methods including, but not limited to, procedural task-trainers, physical models, computer-based patients, high-fidelity full-body simulators, and standardized patients (lay people who are specifically trained to portray patients). In particular, high-fidelity simulator exercises are increasingly being incorporated into both pre-clinical (1, 4, 9) and clinical curricula (1, 4). Although high-fidelity simulators are used for teaching clinical skills in the pre-clinical setting, there is limited data regarding the efficacy at this early stage of training (9–12). Instead, most medical school studies of simulation have focused on the effectiveness of teaching clinical skills to students during their clerkships (13–19), mainly in their fourth year.

Our study included students who were enrolled in ‘Introductory Clerkship’, a pre-clinical course for medical students transitioning from their second year into their third year. This course takes place in June at our medical school, immediately before students begin their clinical clerkships. It is a 1-month ‘crash’ course teaching an array of practical skills that students will need during their upcoming clinical rotations. The course addresses common chief complaints including dyspnea, abdominal pain, chest pain, and altered mental status (AMS). For each chief complaint, students attend a 1-hour lecture followed by a 2-hour small group, interactive case-based session. Faculty members from multiple disciplines facilitate small group sessions. Using AMS as an example, outlines of the 1-hour lecture and the corresponding small group cases are presented in Table 1 and 2, respectively. Although these sessions teach practical clinical skills, we were concerned that there existed a translational gap for the students between their ability to understand and discuss these clinical problems and actually implementing these skills at the bedside of an acutely ill patient. We felt the learning experience could be greatly enhanced if students had the opportunity to practice these skills in ‘real’ clinical encounters with the use of simulation.

**Table 1:** Altered Mental Status: Lecture Outline

Recognizing altered mental status (AMS) Defining terms: delirium, dementia, coma Categorizing AMS etiologies (systemic, focal brain, vs. diffuse brain pathology) Overall approach to the altered patient Assessing airway, breathing and circulation (ABCs) Utilizing vital signs to focus differential diagnosis Temperature, blood pressure, heart rate, respiratory rate Oxygen saturation, finger stick Key history and physical exam findings Focused neurological exam Utilizing diagnostic testing Case presentations discussing the approach to the agitated AMS patient Case presentations discussing the approach to the mentally depressed AMS patient

**Table 2:** Altered Mental Status: Small Group Cases

Case #1: 85-year-old nursing home patient with depressed mental status Case #2: 20-year-old student agitated outside of a rock concert Case #3: 37-year-old subtle mental status change and headache Case #4: 54-year-old female in a coma state
Format: Each case discussion reviews initial approach at the bedside, including vital sign assessment, essential history and physical exam elements, initial treatment, differential diagnosis, and appropriate diagnostic testing. Sessions are 2 hours with one faculty facilitator and 10–15 medical students. Emphasis was placed on the first two cases which were designed by a study author (Author #1).

We hypothesized that the addition of high-fidelity simulation for pre-clinical students who have not yet started their clinical rotations would positively impact knowledge acquisition, perceived competence in their clinical approach, and comfort caring for patients. In addition, we hypothesized that students who participated in the simulation would rate the overall curriculum as more useful.

## Methods

### Study population

Second-year medical students, prior to entering their third year, who were enrolled in the Introductory Clerkship course were eligible to participate in this study. A total of 101 students were eligible to participate in June 2010, and 101 students were eligible in June 2011. A simulation exercise was developed as a pilot for the AMS component of the course.

The Clinical Curriculum Coordinator recruited eligible medical students. Students were told that it was a voluntary simulation exercise. Students were also told that the simulation would not have any impact on their course grade and that any data collected would not be part of their formal course records or provided to the course director. Students who wanted to participate had to sign up in person for an available simulation session. Student participation was on a first come, first served basis, completely voluntary, and due to resource constraints, limited to 40 students per year, and 10 students in any given 1-hour simulation session. The study was approved by the WCMC IRB as educational research, and written informed consent was waived.

### Simulation sessions

The simulation sessions took place on a high-fidelity, full-body simulator (METI HPS – Medical Education Technologies, Sarasota, FL) in our Clinical Skills Center, in a room specifically designed for simulation sessions. The two AMS cases developed for the session were: 1) a patient with an overdose of medications and alcohol who has respiratory depression and hypoglycemia; and 2) an agitated head trauma victim who is post-ictal after a seizure and then has another seizure (Table 3). Students were split into two groups. Four to five students were participants in the case while the other students were observers. The students then switched roles for the second scenario. All students participated in a debriefing session (described below).

**Table 3:** Altered Mental Status: Simulation Cases

**Case #1**: 25-year-old unconscious female patient.
Case Summary: Patient has altered mental status from an overdose of alcohol and medications. The patient has a bag full of empty pill bottles and an empty vodka bottle. There are superficial lacerations on the patient's arms. Students must recognize that this is a possible suicide attempt with a medication overdose. Systematic evaluation of the patient will reveal that the patient is inadequately breathing requiring bag valve mask ventilation, and that the patient is hypoglycemic requiring intravenous dextrose.
Case duration: 10–12 minutes; 12–15 minute debriefing

**Case #2**: 22-year-old male with altered mental status. Patient is post-ictal after a seizure.
Case Summary: At the beginning of the scenario, the patient has altered mental status because he is post-ictal after a seizure. During the encounter, the patient has a tonic–clonic seizure. Students must treat the seizure. If the students perform a careful physical exam, they will find a posterior scalp hematoma. They should then order a head CT for a possible intracranial bleed.
Case duration: 10–12 minutes; 12–15 minute debriefing

Complete descriptions of these cases are available in an online supplement.

### Facilitators

Sessions were conducted by three faculty members (including Authors #1 & #3). The three faculty members are all clinician educators with extensive experience with simulation and case-based instruction, and all have taken coursework in simulation. Two are emergency medicine physicians; the third is an internist and Director of the Clinical Skills Center. All simulation sessions were run by the same three faculty members; their roles for running the simulations, role-playing in the cases and debriefing were the same for every session.

### Debriefing

Faculty observed students during the simulation and students then participated in a 15-minute debriefing after each case. During the debriefing, feedback was provided in an ask–tell–ask approach: 1) students were asked to provide self-reflections about perceived strengths and areas of improvement; 2) faculty provided tailored responses which included specific feedback points based on observations; and 3) faculty asked students about the major take-home points based on the debriefing and addressed any additional student questions.

### Post-test and survey design

The post-test and survey were designed to test student knowledge acquisition, perceived competence in their clinical approach to AMS, their comfort caring for an altered patient, and the usefulness of the AMS curriculum. Post-test questions were designed to cover material from the lectures and small group discussions that were then reemphasized in the simulation sessions. After the post-test and survey questions were written, they were field tested by EM resident and attending physicians and Clinical Skills Center faculty until there was consensus about question clarity, meaning, and the correct answers for knowledge-based questions. Eight physicians took part in the field-testing.

### Assessment and outcomes

Both students who did and did not participate in the simulation sessions completed a written post-test (Table 4) to assess knowledge and a survey to assess perceived competence and comfort in their clinical approach to the management of AMS. Students who participated in the simulations took the post-test and survey immediately following the simulation session. The next day, students who participated in the simulation were excused early for lunch, and the remaining students completed the post-test and survey.

**Table 4:** Altered Mental Status: Post-Test

1. An elderly male presents with a depressed level of consciousness. You detect what you believe is alcohol on his breath. In the first 10 minutes of the patient's arrival to the emergency department which of the following would be your lowest priority?
A) Connect the patient to an intravenous catheter and a cardiac monitor
B) Obtain a toxicology screen
C) Assess airway and breathing; supply supplemental oxygen if needed
D) Obtain a rectal temperature
E) Obtain a finger stick
2. An agitated 25 year old presents to the emergency department with sinus tachycardia of 120. All of the following are diagnoses that need to be considered except:
A) Cocaine intoxication
B) Encephalitis
C) Severe hypothermia
D) Post-ictal state after a generalized tonic–clonic seizure
E) Hypoglycemia
3. You (a third year medical student) are alone with an unconscious patient on the medical wards. The following would by a correct sequence for approaching the patient:
A) Airway, Breathing, Circulation Assessment – Assess Responsiveness – Call for Help – Check Glucose
B) Airway, Breathing, Circulation Assessment – Assess Responsiveness–Check Glucose – Call for Help
C) Call for Help – Airway, Breathing, Circulation Assessment – Check Glucose – Assess Responsiveness
D) Assess Responsiveness – Call for Help – Airway, Breathing, Circulation Assessment – Check Glucose
E) Check Glucose – Assess Responsiveness – Call for Help – Airway, Breathing, Circulation Assessment
4. You are now evaluating an elderly unconscious woman. Which of the following bedside diagnostic tests would be least likely to reveal the cause of the patient's altered mental status?
A) Finger stick
B) Rectal temperature
C) Pulse oximetry
D) Hematocrit
E) Pupil exam
5. You are now evaluating an elderly unconscious woman. Which of the following physical exam findings is not a classic set of findings for an etiology of altered sensorium?
A) Pin point pupils
B) Sweaty skin and tachycardia
C) Calf swelling and tachycardia
D) Asterixis
E) Dry skin and full bladder
6. Which of the following causes of altered mental status is most rapidly reversible?
A) Urosepsis
B) Cocaine intoxication
C) Hepatic encephalopathy
D) Opiate overdose
E) Intracranial bleed
7. Which of the following is an incorrect action for a patient who is actively having a seizure?
A) Insert an oral airway
B) Check glucose (finger stick)
C) Administer a benzodiazepine (e.g., lorazepam)
D) Provide supplemental oxygen
E) Keep the patient away from sharp objects
8. Which of the following is true about the history in a patient with altered mental status?
A) Treatment should not be started without a complete medical history
B) The patient's medical records are not typically helpful
C) A history of recent medication adjustments should be elicited
D) Patients with altered mental status generally provide an accurate and detailed history
E) Knowledge of the patient's baseline mental status is generally not be helpful
Answer key: 1) B 2) C 3) D 4) D 5) C 6) D 7) A 8) C

Knowledge acquisition was measured using the eight-question knowledge-based post-test. Perceived competence was measured using the following questions: ‘I can articulate a differential diagnosis for a patient with altered mental status;’ ‘I understand what initial diagnostic tests are needed in the evaluation of a patient with AMS;’ and ‘I understand what interventions are useful in the first few minutes in the treatment and evaluation of a patient with AMS.’ Comfort caring for an altered patient was measured by the following question: ‘I feel comfortable in my overall approach to a patient with AMS.’ Students were also asked how useful they found the AMS curriculum. Questions on the survey used a five-point Likert scale.

### Statistical analysis

Analyses were conducted using Stata 12.0 (StataCorp, College Station, TX). Results are presented as proportions or medians (with interquartile ranges). Comparisons between students who did and did not participate in simulation sessions were made using Chi-square, Fisher's Exact, and Kruskal Wallis tests, as appropriate. A *p* value of <0.05 was considered statistically significant.

## Results

The post-test and survey were completed by 154 (76%) of the 202 students enrolled in the course. Baseline demographics for the study participants were as follows: 53 males and 48 females with a mean age of 24.0 in 2010, and 55 males and 46 females with a mean age of 23.6 in 2011. The simulation session was attended by 65 (42%) of the 154 students. Among these 65 students, 48 attended both the lecture and small group sessions, while 17 attended either the lecture or the small group session. Of the students who did not attend the simulation, 58 (38%) attended both the lecture and small group and 16 (10%) attended either the small group or the lecture but not both. Fifteen students (10%) did not attend any of the sessions.

**Fig. 1:**
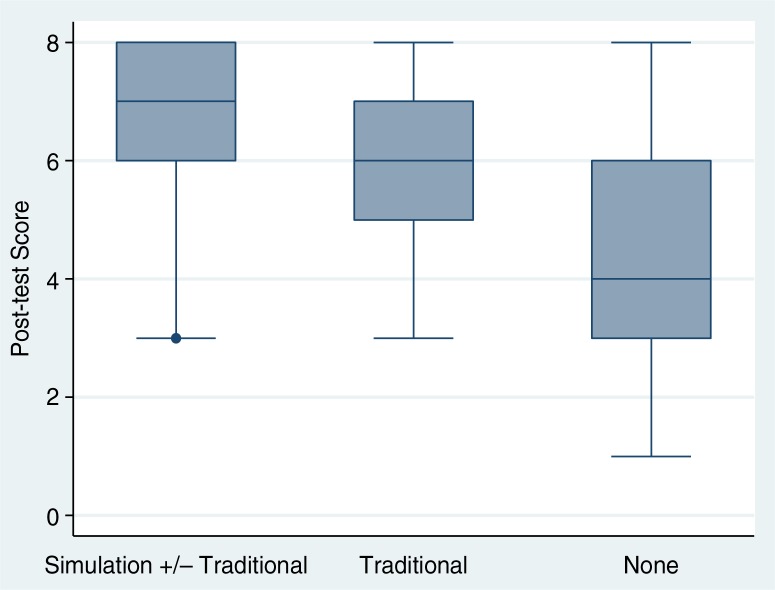
Post-test median scores by type of instruction. Median post-test scores with interquartile range and range of values for students who participated in the simulation session (with or without lecture or small group case-based sessions), students in the traditional curriculum (in lecture or small group case-based sessions only), and students who did not attend any sessions.

The median post-test score was 6 (IQR, 5–7). Figure 1 displays post-test scores for students who attended a simulation session, for students who attended only traditional sessions, and for students who did attend any of the AMS sessions. Post-test scores were higher in students who attended the simulation session compared with students who did not (7 [IQR, 6–8] vs. 6 [IQR, 4–7]; *p*<0.001). Students who did not attend any AMS session had a median score of 4 (IQR, 3–6), which was significantly lower than students attending any sessions (*p*<0.001).

Students who attended the simulation session, compared to students who attended traditional sessions only or no sessions, were more likely to report that they could articulate a differential diagnosis for an AMS patient, know what initial diagnostic tests are needed, and what interventions are needed in the first few minutes for an AMS patient as well as more likely to feel comfortable in their approach to an AMS patient. Students who attended the simulation session were also more likely to rate the overall AMS curriculum as useful (Table 5).

**Table 5:** Altered Mental Status: Post-Exercise Survey

	Attended simulation (*n*=65), %	No simulation (*n*=89), %	*p*
After the Intro to clerkships course (% reporting yes):			
I feel comfortable in my overall approach to a patient with AMS.	58	42	0.05
I can articulate a differential diagnosis for a patient with altered mental status.	66	47	0.03
I understand what initial diagnostic tests are needed in the evaluation of a patient with AMS.	74	53	0.01
I understand what interventions are useful in the first few minutes in the treatment and evaluation of a patient with AMS.	79	56	0.003
Overall, I found the curriculum on AMS useful.	94	61	<0.001

## Discussion

In this study, we found that the addition of a simulation exercise to our traditional AMS curriculum (lecture and case-based small group discussions) led to improved performance on a knowledge-based post-test, to higher student perceived competence in their clinical approach to the altered patient, and increased student comfort caring for an AMS patient. Medical students who attended the simulation sessions found the overall AMS curriculum more useful. Our results are highly encouraging demonstrating that SBME can be a useful adjunct tool for teaching practical clinical skills even before students enter their clinical clerkships. Although students’ perception of their own clinical competence or comfort may not translate into clinical skill acquisition, helping students become confident and comfortable with clinical medicine at an early stage of training (i.e., prior to even entering the wards) may make their transition to the care of real patients less stressful and more educational.

We incorporated this simulation exercise as a pilot into our AMS curriculum to provide students the opportunity to apply key concepts from the lecture and small group case discussions. Students were able to practice skills that are difficult to teach effectively in the lecture or classroom environment. For example, to be successful in the simulations, students had to assess the patient's airway and breathing, had to systematically assess the patient in order to find any life-threatening diagnoses, had to integrate multiple pieces of clinical data to generate a differential diagnosis, and had to prioritize needed diagnostics and treatments. Additionally, students in our simulation exercise practiced working as a team in the management of a critically ill patient and had the opportunity to play the role of a physician who had to discuss their patient's case with a family member or consultant. Students became immersed in the clinical cases and reported that they felt the ownership and pressure of dealing with a ‘real’ critically ill patient. Simulation provides a realistic and interactive learning environment that promotes practicing real-time clinical judgment, communication, and hands-on skills (14). It has been estimated in the educational literature, that partaking in a simulated exercise where students practice what they have learned could result in up to 75% retention, compared to retaining 20% of what they hear in a lecture and 50% of what they learn engaged in a discussion (20). As students are immersed in the clinical experience of simulation, they are gaining experience through auditory, visual, and tactile stimuli and this level of engagement creates a different learning environment than the classroom.

SBME has been shown to be highly effective in many clinical arenas including improving patient safety (21, 22), long-term retention of resuscitation guidelines (23), the development of procedural skills in different disciplines (24–27), and operative skills (28–30). However, little has been documented about the impact of simulation on early clinical skills development in pre-clerkship medical students.

Despite lack of evidence of effectiveness at this early stage, a number of medical schools are incorporating high-fidelity simulators into the pre-clinical years to teach clinical skills (1, 4, 9). Our study adds to this budding literature by beginning to evaluate the effectiveness of incorporating simulation into the curriculum for pre-clinical students. Gordon, Brown, and Armstrong reported on a small pilot project where a myocardial infarction simulation case was used to teach cardiovascular physiology to first year medical students, and found that students who participated in the simulation had better scores on a knowledge-based test at one year compared to controls. This study had a small sample size (*n*=22 students) and was not randomized, and participating students were enrolled as a convenience sample (9). Halm, Lee, and Frank studied the impact of a simulation exercise involving an overdose patient for second-year medical students; they found an improvement on a knowledge-based test when simulation was added to a problem-based learning session (10). This small study (*n*=50 students) did not include a control group. Goodrow, Rosen, and Wood explained the role of simulation at their two medical schools (4). Specifically, they described the incorporation of simulation sessions into pre-clinical courses such as pharmacology, physiology, and introductory clinical skills courses. In their curriculum, simulated cases are utilized to make these foundation courses less abstract and more practical, but they did not study the effectiveness of their new simulation curriculums. Additionally, at one medical school, simulation penetration into the pre-clinical curriculum was reported as slow, and student participation had been mostly optional (4). Our study contributes to this literature with a larger group of students and with an intervention group (students electing to participate in a simulation session) and a control group (students participating in the traditional curriculum or no sessions at all).

Most other studies in pre-clinical coursework focus on either specific diagnostic skill acquisition or teaching physiology or pharmacology rather on learning a general approach to a chief complaint, as in our study. For example, one study found a positive impact of simulation on specific cardiac and pulmonary diagnostic skills for first year medical students (11) and another on the physiological effects of anesthetic agents on hemodynamic parameters (12). As described earlier, most simulation medical school interventions like ours have focused on third- and fourth-year students in clerkships like emergency medicine (13–15, 18, 19), surgery (16), and anesthesia (17).

Unlike many previous studies (13–15), the primary intent of this study was to assess whether simulation had an additive benefit to a traditional curriculum rather than as a replacement to existing formats such as lectures or problem-based learning (PBL) sessions. Our intention was to use the simulation experience for experiential learning (6) and deliberate practice (7, 8) as a method for reinforcing key teaching points that were emphasized in the lectures and PBL cases. The overall goal was for the simulation to improve long-term knowledge retention and help the students develop real clinical skills. For example, the importance of assessing airways and breathing, obtaining a full set of vital signs, and checking for hypoglycemia were key teaching points and the simulations allowed the students the opportunity to translate this knowledge into physical actions in a clinical situation.

### Limitations

The limitations of this study include lack of a pre-test to measure baseline differences across the groups. We were also unable to assess for changes following the educational curriculum or to adjust for baseline knowledge or other confounders. Although it is possible that students had baseline differences in knowledge, all students received the same 2-year pre-clinical medical education at the time of the study. This study also lacks measurement or observation of applied skills, a limitation that we plan to address in future work.

Additionally, due to resource constraints, this pilot study was limited in the number of students able to participate each year (*n*=40 students). Students who signed up to participate in the simulation exercise may be a more motivated group of students, thus biasing the observed results. We believe, however, that this potential bias is minimized by the fact that many students who did not participate in the simulation session expressed disappointment in not being able to do so. Additionally, the positive results could be a result of the students getting ‘extra’ educational time and not necessarily the simulation itself. Although certainly possible, the ‘extra’ teaching is the point of the study – we wanted to measure whether this supplemental teaching exercise would improve students’ retention of material and make them more comfortable in their clinical skills.

Finally, the post-test and survey were designed by field-testing questions considered to be relevant by the study investigators. Neither were validated or assessed for reliability. Future work will use structured methods for instrument development.

### Conclusion and Future Directions

High-fidelity simulation may be a potentially effective way of teaching clinical skills to medical students during pre-clinical curriculum. Further study is needed to assess how best to incorporate it. Future research will determine whether use of simulation is effective for teaching other major chief complaints (e.g., dyspnea, chest pain) in a pre-clinical setting and will assess improvements in knowledge using a pre-test/post-test design. Most importantly, future research should attempt to assess whether this type of simulation session for a pre-clinical student actually improves future clinical performance in these topic areas and patient outcomes.
